# Real-World Data Quality Framework for Oncology Time to Treatment Discontinuation Use Case: Implementation and Evaluation Study

**DOI:** 10.2196/47744

**Published:** 2024-03-06

**Authors:** Boshu Ru, Arthur Sillah, Kaushal Desai, Sheenu Chandwani, Lixia Yao, Smita Kothari

**Affiliations:** 1 Center for Observational and Real-world Evidence (CORE) Merck & Co, Inc West Point, PA United States

**Keywords:** data quality assessment, real-world data, real-world time to treatment discontinuation, systemic anticancer therapy, Use Case Specific Relevance and Quality Assessment, UReQA framework

## Abstract

**Background:**

The importance of real-world evidence is widely recognized in observational oncology studies. However, the lack of interoperable data quality standards in the fragmented health information technology landscape represents an important challenge. Therefore, adopting validated systematic methods for evaluating data quality is important for oncology outcomes research leveraging real-world data (RWD).

**Objective:**

This study aims to implement real-world time to treatment discontinuation (rwTTD) for a systemic anticancer therapy (SACT) as a new use case for the Use Case Specific Relevance and Quality Assessment, a framework linking data quality and relevance in fit-for-purpose RWD assessment.

**Methods:**

To define the rwTTD use case, we mapped the operational definition of rwTTD to RWD elements commonly available from oncology electronic health record–derived data sets. We identified 20 tasks to check the completeness and plausibility of data elements concerning SACT use, line of therapy (LOT), death date, and length of follow-up. Using descriptive statistics, we illustrated how to implement the Use Case Specific Relevance and Quality Assessment on 2 oncology databases (*Data sets A and B*) to estimate the rwTTD of an SACT drug (*target SACT*) for patients with advanced head and neck cancer diagnosed on or after January 1, 2015.

**Results:**

A total of 1200 (24.96%) of 4808 patients in Data set A and 237 (5.92%) of 4003 patients in Data set B received the target SACT, suggesting better relevance of the former in estimating the rwTTD of the target SACT. The 2 data sets differed with regard to the terminology used for SACT drugs, LOT format, and target SACT LOT distribution over time. Data set B appeared to have less complete SACT records, longer lags in incorporating the latest data, and incomplete mortality data, suggesting a lack of fitness for estimating rwTTD.

**Conclusions:**

The fit-for-purpose data quality assessment demonstrated substantial variability in the quality of the 2 real-world data sets. The data quality specifications applied for rwTTD estimation can be expanded to support a broad spectrum of oncology use cases.

## Introduction

### Background

The importance of real-world evidence drawn from real-world data (RWD) is widely recognized in oncology research [1-5]. Over the past decade, federal legislation and incentives promoting the secondary use of RWD in the United States [6-8], coupled with advances in health information technology, have resulted in an explosion of RWD sources and a complex RWD ecosystem [1]. However, this rich data landscape can also pose challenges in identifying fit-for-purpose RWD to meet biopharma research needs.

Two key obstacles to identifying high-quality data are the fragmentation of RWD sources and the lack of interoperable data quality standards. These obstacles are particularly pertinent in the United States, where progress is slow in reaching full interoperability of data sourced from thousands of providers who customized their electronic health record (EHR) systems from solutions provided by >40 different EHR software vendors [9]. Therefore, adopting validated systematic methods for evaluating data quality is important for research leveraging RWD [10-12].

In 2016, an expert panel proposed the concepts of *conformance*, *completeness*, and *plausibility* as 3 categories (with subcategories) to describe the intrinsic data quality of EHR databases and to serve as a framework for assessing data quality that could then be verified (with organizational data) or validated using an accepted gold standard [13]. Several working groups and authors have applied these terms or proposed others for defining research data quality [14-16], and multiple initiatives in the United States, both public and private, have developed frameworks and tools to evaluate and improve the quality of EHR data sets [17-21] and to implement model-driven, quantitative approaches to address RWD completeness and plausibility issues [22-25]. However, there is no single RWD source that can fit the needs of all studies, and the selection of RWD to support an individual use case must also consider data relevance and measurement thresholds in addition to data quality.

### Objective

In a previous study, we introduced the Use Case Specific Relevance and Quality Assessment (UReQA) framework, an RWD quality framework that combines both the data quality and the relevance aspects of assessing RWD, with the goal of developing data quality assessment specifications tailored to use cases [3]. In this study, we aimed to implement this framework in the use case for estimating real-world time to treatment discontinuation (rwTTD) in oncology. Our work had two main components: (1) to design comprehensive data quality assessment checks for estimating rwTTD for a systemic anticancer therapy (SACT) and (2) to illustrate how these quality checks can be used to evaluate EHR-derived RWD products.

We selected rwTTD as the first use case to implement the UReQA framework because of its high utility as a pragmatic real-world effectiveness end point for continuously administered SACTs (such as immunotherapies) and its known correlation with overall survival [26-28]. Moreover, the estimation of rwTTD requires information on medication use patterns, mortality, and follow-up. These data elements are foundational to outcomes research. Therefore, implementation of the rwTTD use case can be expanded to other use cases in or beyond oncology, as well as different data sources, such as claims databases.

## Methods

### Ethical Considerations

This study used 2 commercially licensed deidentified structured secondary data sources accessible to the study team. It was exempted from institutional review board review because of the following: (1) each data source contains a significant level of protection against the release of personal information to outside entities and (2) the use of such databases presents the lowest risk to potential subjects because the analysis involves only anonymous data; hence, conducting the study will not place the subjects at risk.

### Study Overview

This study comprised four main steps: (1) conceptual definition of the rwTTD use case; (2) mapping of the rwTTD use case definition to RWD elements (operational definition); (3) identifying data quality checks for the required data elements to determine rwTTD for an SACT, designated the “target SACT”; and (4) implementing the UReQA framework [3] in assessing the RWD fitness for estimating rwTTD. The data quality assessment was undertaken on 2 US EHR-based oncology databases for estimating rwTTD for a target SACT, an immunotherapy drug that is administered intravenously in advanced-stage head and neck cancer (HNC). The targeted SACT received approval in 2016 for the treatment of previously treated advanced HNC and in 2019 for its use as a first-line therapy in advanced HNC. The focus of this study is on designing data quality assessment methods that are tailored for specific use cases, rather than calculating rwTTD for a particular medication. Therefore, we mask the name of the actual drug product.

### Step 1: Conceptual Definition of the rwTTD Use Case

The end point, rwTTD, is defined as the length of time from initiation to discontinuation of a medication *([date of last recorded dose – date of first recorded dose] + 1 d*), with discontinuation defined as the date of the last dose if a patient died during therapy or initiated a new treatment or if there is a gap of ≥120 days between the last recorded dose and last recorded activity in a data set. Patients who do not meet the discontinuation criteria are censored at the last medication use [26-28].

### Step 2: Mapping of the rwTTD Use Case Definition to RWD Elements

Owing to the variations in data element definition and data structures between real-world EHR databases, we need to operationalize the concept of rwTTD by deconstructing its definition and mapping it to four sets of required data elements that are commonly available from oncology EHR–derived data sets: (1) SACT, (2) line of therapy (LOT) specifying the regimen names and sequence of current treatment in the treatment plan [29,30], (3) mortality status, and (4) follow-up time, as summarized in Table 1. Although SACT, mortality status, and follow-up time are often recorded directly as procedure, prescription, and administrative events in raw EHR databases, the LOT was often derived from raw EHR by the algorithm.

**Table 1:** Required data elements for determining real-world time to treatment discontinuation (rwTTD) for a systemic anticancer therapy (SACT) drug in a specific line of therapy (LOT).

Operational steps to ascertain rwTTD and type of data category		Commonly used data elements
**Identify records of the drug of interest**		
	SACT drug	Drug_name, NDC^a^, HCPCS^b^ code, RxNorm code
	SACT administration	Drug administration date^c^
	SACT order	Drug order date^d^
**Identify discontinuation date from subsequent LOT start date**		
	LOT	LOT name^e^
	LOT	LOT number
	LOT	LOT start date
	LOT	LOT end date
**If no subsequent LOT, identify discontinuation date from patient death record during treatment**		
	Mortality status	Vital status or date of death
**If no date of death, identify discontinuation date by last follow-up date subheading**		
	Last follow-up	Date of last follow-up^f^

^a^NDC: National Drug Code.

^b^HCPCS: Healthcare Common Procedure Coding System.

^c^The drug administration date is defined as the date of receiving medication at a health care facility as a medical service, often applicable to an intravenous drug.

^d^The drug order date is defined as the order date for drugs used at home, often applicable to an oral drug.

^e^The LOT name is determined by the combination of SACT drugs administered or ordered from the LOT start to end dates.

^f^The date of last follow-up is defined as the last documented clinic visit or procedure in the electronic health record.

We defined SACT as any systemic anticancer medication received by the patient, documented as given either by a health care provider at the site of care (eg, by infusion), with the date defined as the “administration” date, or as a prescription to take or apply at home, with the date defined as the “drug order” date. The number of refills (or alternative data elements such as days of supply or expected medication end date) was used to determine the last use of oral drugs (Table 1).

LOT was defined as the sequence of the SACT regimens prescribed for an individual patient, as previously described in detail [29,30]. In brief, the first LOT (line 1 [1L]) begins with the first SACT initiated after a study index date (often the advanced or metastatic cancer diagnosis date), and any other drug introduced within the next 28 days is considered part of that LOT [29]. We defined the start of a new LOT when a new SACT not belonging to the prior LOT was introduced or if a new SACT was initiated after a ≥120-day gap in therapy.

Because the target SACT was administered intravenously, we omitted 2 tasks applicable only to oral target SACTs: the check of patient numbers with target drug order date after the index date (Multimedia Appendix 1, task 6 [13]) and the check for distribution of gaps between drug order dates (Multimedia Appendix 1, task 9).

The patient mortality status was determined based on the recorded dates of death. For patients who were still alive at data cutoff, the date of the last follow-up was defined as the last documented clinical activity date in the EHR (Table 1).

### Step 3: Identifying Data Quality Checks for Required Data Elements

For each of the required data elements, we identified corresponding verification checks to assess data quality at both the variable level and the cohort level. A total of 20 data quality checks (tasks) were identified and categorized into the quality dimensions of conformance, completeness, and plausibility, as per the harmonized data quality assessment terms and framework developed by Kahn et al [13] (Multimedia Appendix 1). Our goal in creating these tasks was to develop a comprehensive toolbox for assessing data quality for the rwTTD use case. However, when adapting them to a specific RWD database and a SACT drug of interest, not every task and check would be necessary. For example, the checks for LOT, mortality, and follow-up are not needed if a data set already provides the reason for discontinuation and censored status for each drug exposure. In addition, tasks 3-5 were applicable to cancer therapies received in hospitals or clinics as intravenous or infusion procedures, whereas tasks 4-9 were dedicated to oral cancer therapies that were mostly self-administrated at home. As tracking the actual time patients took oral therapies was infeasible, researchers examined days supply and refill records to estimate the drug exposure period. Therefore, when investigating the rwTTD of an oral SACT drug, it is necessary to check the completeness of these oral therapy–specific data elements (task 7).

### Step 4: Implementing the rwTTD Use Case for Assessing 2 RWD Sets

#### Data Set Preassessment

We followed the preassessment step in UReQA [3] to identify 2 anonymized, commercially available US real-world oncology databases, designated as *Data set A* and *Data set* *B* in this report, which included patients with advanced (metastatic or unresectable, recurrent) HNC. Both databases contained data elements sourced from structured and unstructured information captured within health care providers’ EHR systems as part of routine cancer care.

#### Cohort Selection and Patient Characteristics

Data set A was commercialized and included patients with advanced HNC, whereas Data set B included patients with all stages of HNC. To align the 2 patient populations as having advanced HNC, we restricted Data set B to the subset of patients with HNC and a record of the American Joint Committee on Cancer stage IV and *International Classification of Diseases* (ICD), *revision 9 or 10* (ICD-9 or ICD-10) code for metastatic tumor (ICD-9 codes 196.x, 197.x, and 198.x and ICD-10 codes C76.x, C77.x, and C78.x). The distributions of the patient characteristics were then tabulated for the 2 data sets.

#### Data Elements Harmonization

In Data set A, the names of SACT medications were harmonized from clinic formulary information and medical service records to standard generic drug names in a commercial drug database along with drug category information. In Data set B, all medication records in the raw EHR data were harmonized into the RxNorm code [31]; however, drug category information was not available. To harmonize all SACT medication in Data set B, we retrieved the RxNorm codes for generic names of all SACT medications using the RxNav software developed by and available from the US National Library of Medicine [32].

The LOT information was previously derived by both data providers but was presented differently in the 2 data sets. In Data set A, the LOT table provided the LOT number, LOT regimen name, LOT start date, and LOT end date, with a flag indicative of maintenance therapy, as appropriate. Instead, Data set B included only the LOT number and LOT start date. Therefore, to evaluate the LOT information in Data set B, we indirectly deduced the end date of each LOT as the date before the start of the next LOT or as the data cutoff date for the last LOT in the data set. Then, all individual SACT medications administered or ordered between the LOT start and end dates were combined to serve as the LOT regimen name. This approach was a necessary but imperfect solution because the LOT end date and the LOT regimen name should ideally be generated using a more rigorous algorithm [29,30].

The date of death was provided at the month and day levels in Data set A, whereas in Data set B, the death date was aggregated by year. Given the relatively short length of survival of many patients with advanced HNC [33-36], the allocation of death dates by year was not sufficiently granular for accurate rwTTD calculation; better precision (ie, month of death) would be needed for accurate rwTTD calculation. Consequently, quality assessment tasks related to mortality variables were omitted (task 17) for Data set B.

#### Reporting the Verification Results

Descriptive statistics were used to summarize the results of implementing rwTTD data quality checks on Data sets A and B. We used frequencies to summarize categorical variables and mean (SD) and median (IQR or range) to summarize continuous variables. The study index date was the date of first advanced HNC diagnosis, and the cutoff date was November 25, 2019.

All analyses were conducted using SAS Studio release 3.8 (Basic Edition; SAS Institute, Inc).

## Results

### Patient Characteristics

Data set A included 7366 patients with advanced HNC, and we identified 11,386 patients in Data set B with advanced HNC. The median patient age at the first advanced HNC diagnosis was 65 (IQR 58-72) years in Data set A and 61 (IQR 54-68) years in Data set B, and the percentages of male individuals were 74.16% (5643/7366) and 69.97% (7967/11386), respectively (Table 2), similar to the HNC population data from the United States [33,37].

**Table 2:** Baseline characteristics of patients with advanced head and neck cancer (HNC) included in 2 data sets under evaluation^a^.

Characteristic		Data set A (n=7366)	Data set B (n=11,386)
**Sex, n (%)**			
	Female	1723 (23.4)	3408 (29.9)
	Male	5643 (76.6)	7967 (70)
	Missing or unknown	0 (0)	11 (0.1)
Age at first advanced HNC diagnosis (y), median (IQR)		65 (58-72)	61 (54-68)
**Age at first advanced HNC diagnosis (y), n (%)**			
	<18	0 (0)	31 (0.27)
	18-44	187 (2.53)	688 (6.04)
	45-64	3402 (46.19)	5955 (52.3)
	65-88	3777 (51.28)	4111 (36.11)
	≥89	0 (0)	6 (0.05)
	Missing or unknown	0 (0)	595 (5.23)
**Race or ethnicity, n (%)**			
	American Indian or Alaska Native	N/A^b^	40 (0.35)
	Asian	103 (1.4)	165 (1.45)
	Black or African American	487 (6.61)	1250 (10.98)
	Hispanic or Latino	13 (0.18)	0 (0)
	Native Hawaiian or other Pacific Islander	N/A	6 (0.05)
	White	4939 (67.05)	9239 (81.14)
	Missing	650 (8.82)	686 (6.02)
	Other race	1174 (15.94)	0 (0)
**AJCC^c^ stage at first HNC diagnosis, n (%)**			
	0	2 (0.03)	28 (0.25)
	I	419 (5.69)	603 (5.3)
	II	505 (6.86)	542 (4.76)
	III	929 (12.61)	798 (7.01)
	IV	4330 (58.78)	4978 (43.72)
	Missing or unknown	1181 (16.03)	4437 (38.97)
**Year of first advanced HNC diagnosis, n (%)**			
	Before 2006	0 (0)	1245 (10.9)
	2006-2009	0 (0)	1537 (13.5)
	2010-2012	1068 (14.5)	2721 (23.9)
	2013-2018	5435 (73.8)	5577 (49.0)
	2019 or later	863 (11.7)	306 (2.7)

^a^Percentages may not add up to 100% because of rounding.

^b^N/A: not applicable.

^c^AJCC: American Joint Committee on Cancer.

### SACT Data Checks

Overall, 75.91% (5592/7366) and 38.74% (4411/11386) of the patients in Data sets A and B, respectively, had a recorded drug administration or drug order for any SACT (Table 3, task 1). A complete start date (y, mo, and d) was recorded for all SACT administrations and orders in both data sets (Table 3, tasks 4 and 8).

**Table 3:** Data quality assessment of SACT^a^ administration and order records after the advanced HNC^b^ diagnosis^c^.

SACT data quality checks			Data set A		Data set B
Task 1: patients with any SACT drug administration or order record after the advanced HNC diagnosis date, n (%)^d^			5592 (75.9)		4411 (38.7)
**Task 2: SACT drug records with missing drug identity (name and code) information**					
	Value, n (%)	0 (0)		0 (0)	
	Normalization of medication name	Normalized generic name		RxNorm ingredient level	
Task 3: patients with target SACT administration date after the advanced HNC diagnosis date, 2015 onward, % (n/N)^e^			24.96 (1200/4808)		5.92 (237/4003)
Task 4: SACT drug administration records with complete administration date, n (%)			425,505 (100)		37,662 (100)
Task 5: gap (in d) between the target SACT drug administration dates, median (IQR; range)			21 (21-21; 1-113)		21 (11-21; 1-824)
Task 6: patients with target SACT order date after the advanced HNC diagnosis date			N/A^f,g^		N/A^g^
Task 7 SACT drug order records with complete days supply and refill information, n (%)			1732 (53.4)		N/A^h^
Task 8: SACT drug order records with complete order date, n (%)^i^			3241 (100)		8380 (100)
Task 9: distribution of gaps (in d) between target SACT drug order dates, normalized by days supply, refill, and cancelation record			N/A^g^		N/A^g^

^a^SACT: systemic anticancer therapy.

^b^HNC: head and neck cancer.

^c^Drug *administration* refers to drugs administered by health care providers at the site of care, whereas drug *order* refers to prescriptions for drugs used at home.

^d^Task 1 was applied to the full data sets, including 7366 and 11,386 patients in Data sets A and B, respectively.

^e^Task 3 was applied for patients with the first advanced HNC diagnosis on or after January 1, 2015, including 4808 and 4003 patients in Data sets A and B, respectively.

^f^N/A: not applicable.

^g^Tasks 6 and 9 were not conducted because they apply to an oral target SACT.

^h^Information about the number of refills, days supply, or alternative data elements was not available in Data set B.

^i^The total number of drug order records in Data set A (3241) and Data set B (8380) was used as the denominator in task 8.

We determined that 4808 (65.27%) of the 7366 patients in Data set A and 4003 (35.16%) of the 11,386 patients in Data set B had a first advanced HNC diagnosis on or after January 1, 2015, the timeline we applied for the study index date as it covered the key diagnostic and therapeutic timeline of the target SACT (first approved in 2016). A total of 1200 (24.96%) of the 4808 patients meeting this timeline in Data set A and 237 (5.92%) of the 4003 patients meeting this timeline in Data set B had a record of receiving the target SACT (Table 3, task 3).

The median length of the gap between target SACT administrations was 21 days in both the data sets, which aligned with the expected dose schedule for the target SACT (Table 3, task 5). However, the range of the gap was considerably shorter in Data set A (1-113 d) than in Data set B (1-824 d), suggesting incomplete target SACT administration records in Data set B.

For the oral SACT records, Data set A included the number of refills and a flag for canceled medication orders, whereas Data set B did not provide refill information (Table 3, task 7). This could impact the accuracy of calculating rwTTD for an orally dispensed SACT because the drug orders for patients remaining on treatment through refills would not be recorded in the database.

### LOT Data Checks

**Figure 1:**
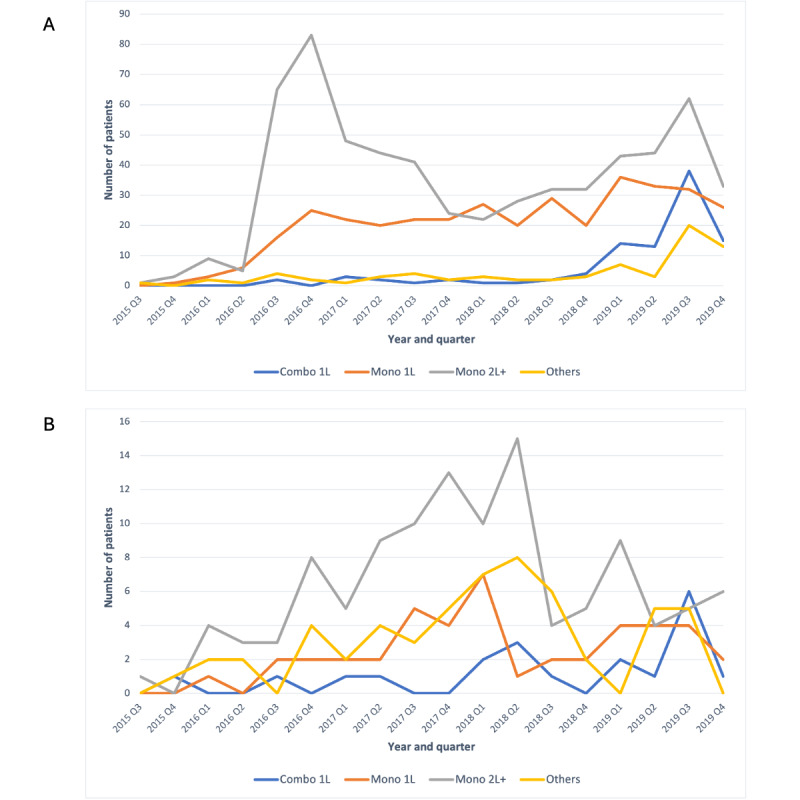
Task 10: number of patients initiating the target systemic anticancer therapy (SACT) by year and quarter in (A) Data set A and (B) Data set B. Note: Y-axis heights in panels A and B differ but were selected to best depict the patient numbers in Data sets A and B. 1L: first-line therapy; 2L+: second-line or later therapy; combo: target SACT in any combination therapy (approved or not approved); mono: target SACT monotherapy; Q1: first quarter; Q2: second quarter; Q3: third quarter; Q4: fourth quarter.

The 2 data sets differed in terms of the target SACT LOT distribution over time. The cumulative frequency of target SACT initiation, including as monotherapy or combination therapy and in any LOT, tended to be greater in later years in Data set A, peaking in the third quarter (Q3) of 2019, than in Data set B, peaking in the first and second quarters of 2018 (Figure 1, task 10). In Data set A, a greater frequency of target SACT initiation as second-line or later monotherapy was consistent with approval timing in this setting (2016), which preceded the first-line approvals (2019). The later time points for second-line or later monotherapy initiation in Data set B suggest the possibility of longer data lags than for Data set A.

In both data sets, we observed the inclusion of patients who initiated the target SACT therapy before the applicable first-line or second-line or later US Food and Drug Administration approval dates. We believe that these are true real-world findings, which do not always correspond to recommended or approved indications, rather than data quality issues.

In Data set B, only 40.3% (4589/11386) of patients had SACT LOT records (Table 4, task 11), which coincides with the finding of lower-than-expected SACT drug administration and order rates (Table 3, task 1).

**Table 4:** LOT^a^ rules for SACT^b^ and mortality information.

Task			Data set A		Data set B				
Task 10: number of patients initiating the target SACT by year and quarter			Figure 1A		Figure 1B				
**Task 11: completeness of LOT information, n (%)^c^**									
	Patients with complete line number		5594 (75.94)		4589 (40.3)				
	Patients with complete line name		5594 (75.94)		N/A^d,e^				
	Patients with complete line start date		5594 (75.94)		4589 (40.3)				
	Patients with complete line end date		5594 (75.94)		N/A^e^				
Task 12: patients for whom the first LOT number after the advanced HNC^f^ diagnosis date was not 1, n (%)^c^			0 (0)		434 (3.81)				
**Task 13: distribution of LOT number at target SACT initiation**									
	Patients who received the target SACT, n		1200		237				
	Line 1, n (%)		481 (40.08)		65 (27.43)				
	Line 2, n (%)		486 (40.5)		92 (38.82)				
	Line 3, n (%)		161 (13.42)		54 (22.78)				
	Line 4, n (%)		46 (3.83)		16 (6.75)				
	Line 5, n (%)		13 (1.83)		10 (4.22)				
	Lines 6-10, n (%)		13 (1.83)		0 (0)				
**Task 14: use of target SACT in 1L^g^ before approval date**									
	**1L monotherapy**								
		Patients who received target SACT, % (n/N)		78.37 (377/481)		67.69 (44/65)			
		First administration date^h^ in database		July 15, 2015		November 10, 2014			
		Cutoff date for the earliest 5% receipt		August 29, 2016		February 4, 2016			
		Cutoff date for the earliest 10% receipt		November 3, 2016		September 23, 2016			
		Cutoff date for the earliest 25% receipt		June 28, 2017		April 27, 2017			
	**Approved 1L combination**								
		Patients who received the target SACT in approved 1L combination, % (n/N)		7.69 (37/481)		6.15 (4/65)			
		First administration date in database		December 18, 2018		July 1, 2019			
		Cutoff date for the earliest 5% receipt		February 18, 2019		N/A^i^			
		Cutoff date for the earliest 10% receipt		April 2, 2019		N/A^i^			
		Cutoff date for the earliest 25% receipt		July 9, 2019		N/A^i^			
Task 15: patients with death record, n (%)^c^			4695 (63.74)		3531 (31)				
Task 16: patients with multiple death records on different dates, n (%)			0 (0)		N/A^j^				
**Task 17: patients with clinical records showing health care activity after death date (n=4695), n (%)**			1497 (31.88)		N/A^j^				
	≥1 d after date of death		1436 (30.59)		N/A^j^				
	≥3 d after date of death		1290 (27.48)		N/A^j^				
	≥7 d after date of death		1002 (21.34)		N/A^j^				
	≥30 d after date of death		79 (1.68)		N/A^j^				

^a^ LOT: line of therapy.

^b^SACT: systemic anticancer therapy.

^c^Tasks 11, 12, and 15 were applied to the full data sets, including 7366 and 11,386 patients in Data sets A and B, respectively.

^d^N/A: not applicable.

^e^Line name and line end date were not available in Data set B.

^f^HNC: head and neck cancer.

^g^1L: first line of therapy after the advanced HNC diagnosis date.

^h^Dates are written as month/day/year.

^i^Not calculated as only 4 patients received the target SACT in a 1L combination LOT.

^j^Only the year of death was available in Data set B.

The LOT start date in both data sets included year, month, and day, and the minimum LOT number started from 1 (first line) after the earliest advanced HNC diagnosis date for all but 3.81% (434/11386) of the patients in Data set B (Table 4, task 12). A line number other than 1 after the advanced HNC diagnosis date suggests that either a definition different from the commonly used definition [29,30] was used or that there was an earlier advanced HNC diagnosis date that was not documented.

In Data set A, 40.08% (481/1200) of patients received the target SACT in first-line therapy and 59.91% (719/1200) in second-line or later therapy, including 13.42% (161/1200) in third-line therapy (Table 4, task 13). In Data set B, 27.4% (65/237) of patients received the target SACT in first-line therapy, and 72.6% (172/237) received it in the second-line or later therapy, with frequent third-line receipt (54/237, 22.8%). Therefore, LOT rules may have been applied differently in Data set A and Data set B.

Complete information about the start date of first-line target SACT drug administration (as both monotherapy and combination therapy) was available for 377+37=414 (86.1%) of 481 patients in Data set A and 44+4=48 (74%) of 65 patients in Data set B (Table 4, task 14). In Data set A, first-line target SACT monotherapy was initiated for the first time in 2015, and approximately 5% of first-line monotherapy initiation dates fell on or before 2016, when the target SACT was approved for second-line or later therapy. Target SACT in combination therapy was first initiated in late 2018, with approximately 25% of the initiation dates falling before the start of Q3 in 2019, shortly after the approval of first-line combination therapy. In Data set B, first-line target SACT monotherapy initiation was first recorded in the fourth quarter in 2014, earlier than in Data set A, and close to 10% of initiation dates occurred before the end of Q3 in 2016. Instead, the approved target SACT combination therapy was first initiated at the start of Q3 in 2019, in line with the approval date for this indication.

### Mortality Data

Among 7366 and 11,386 patients in Data sets A and B, 4695 (63.74%) and 3531 (31%), respectively, had a recorded date of death (Table 4, task 15); and 4427 (60%) and 3093 (27%) patients, respectively, had death records within 3 years after the date of advanced HNC diagnosis. These percentage differences indicate that Data set B may have incomplete mortality records (or a high loss to follow-up).

In Data set A, one-third of patients (1497/4695, 31.88%) with a recorded date of death had clinical records recorded after the death date (Table 4, task 17), with a median of 11 days from the death date to the last activity date. Thus, clinical records could be entered into the health information system after the reported death date, but extreme values (eg, >30 d after the death date) might indicate integrity issues in collecting mortality data. This information was not available for Data set B, in which the dates of death were recorded only by year.

### Follow-Up Data

In Data set A, most patients (5840/7366, 79.28% to 7269/7366, 99.86%) had recorded data for diagnosis, drug records, laboratory results, facility visits, and vital sign measurements (Table 5, task 18). Similarly, in the subset of 7754 patients in Data set B whose advanced HNC diagnosis date was on or after January 1, 2011, the earliest date in Data set A, these data categories were also recorded for most patients (6123/7754, 78.97% to 6893/7754, 88.7%). Records of medical procedures not related to drug administration and genomic testing were not available in Data set A, which could result in inaccurate estimates of follow-up times.

**Table 5:** Unique number of patients and patient-date pairs after the advanced HNC^a^ diagnosis date (task 18): follow-up data for patients with advanced HNC diagnosis on or after January 1, 2011.

Variable	Data set A (n=7366)				Data set B (n=7754)		
	Value, n (%)	Unique patient-date pairs, n	Pairs per patient, n	Value, n (%)		Unique patient-date pairs, n	Pairs per patient, n
Diagnosis	6567 (89.15)	60,178	9.2	6893 (88.9)		370,671	53.8
Drug records^b^	5840 (79.28)	113,948	19.5	6802 (87.72)		269,225	39.6
Laboratory records	6860 (93.13)	179,177	26.1	6403 (82.58)		147,314	23
Facility visit	7269 (98.68)	274,714	37.8	6838 (88.19)		392,175	57.4
Vital sign measurements	7254 (98.48)	233,623	32.2	6123 (78.97)		217,797	35.6
Nondrug medical procedure	N/A^c^	N/A	N/A	6740 (86.92)		390,556	57.9
Genomic test	N/A	N/A	N/A	118 (1.52)		208	1
Biomarker test	440 (5.97)	469	1.1	N/A		N/A	N/A
ECOG PS^d^	5416 (73.53)	100,607	17.7	N/A		N/A	N/A

^a^HNC: head and neck cancer.

^b^Any drug, not just systemic anticancer therapies.

^c^N/A: not applicable.

^d^ECOG PS: Eastern Cooperative Oncology group performance status.

The median frequency of visits (normalized by length between first and last target SACT administration) for patients who received the target SACT was somewhat less in Data set A, varying from 0.05 to 0.12, depending on treatment line, than in Data set B, in which it varied from 0.14 to 0.18 (Table 6, task 19). This might indicate that more clinical activities were recorded in Data set B during treatment.

**Table 6:** Follow-up data for patients with advanced HNC^a^ diagnosis on or after January 1, 2011.

Task			Data set A (n=7366)						Data set B (n=7754)				
			Value, n		Value, median (IQR; range)		Value, mean (SD)		Value, n		Value, median (IQR; range)		Value, mean (SD)
**Task 19: frequency of visits during target SACT^b,c^**													
	1L^d^ combination therapy	101		0.11 (0.07-0.16; 0.02-0.33)		0.13 (0.07)		19		0.17 (0.11-0.24; 0-0.36)		0.17 (0.09)	
	1L monotherapy	358		0.05 (0.05-0.08; 0.01-0.50)		0.07 (0.05)		44		0.18 (0.09-0.22; 0-0.48)		0.16 (0.11)	
	2L+^e^ monotherapy	634		0.06 (0.05-0.10; 0.01-0.95)		0.08 (0.06)		106		0.13 (0.07-0.25; 0-0.75)		0.17 (0.14)	
	All other	104		0.12 (0.09-0.17; 0.02-0.48)		0.14 (0.08)		76		0.14 (0.09-0.21; 0-1.3)		0.17 (0.17)	
Task 20: for patients still alive, gap (in d) from the last target SACT administration and last visit^f^			708		28 (6-187; 0-1118)		128 (199)		167		70 (29-223; 0-1755)		159 (215)

^a^HNC: head and neck cancer.

^b^SACT: systemic anticancer therapy.

^c^Frequency defined as number of visits between the first and last target SACT administration dates within the same LOT number and name, divided by number of days between the last and first target SACT administration.

^d^1L: first line of therapy after the advanced HNC diagnosis date.

^e^2L+: second-line or later therapy.

^f^Limited to patients who (1) were still alive ≥180 days after last receipt of target SACT and (2) received last dose of target SACT ≥180 days before data cutoff on November 25, 2019 (thus on or before May 29, 2019).

## Discussion

### Principal Findings

This study identified 20 data quality assessment tasks for the use case of estimating the rwTTD of an SACT. By executing the 18 tasks pertinent to the intravenously administered target SACT, we demonstrated that the UReQA framework for the rwTTD use case can be implemented to generate descriptive summary statistics and charts. These visualizations provide additional insights into the relevance and quality of 2 US EHR-based oncology RWD. The approach is generalizable to implement for other SACT and databases.

Both data sets in the evaluation provided all the required data elements; however, verification checks revealed that Data set B might not be suitable for analyzing rwTTD for the target SACT because (1) the large decrease in patient receiving the target SACT in recent years suggests longer lags in incorporating the most recent data and (2) the completeness and plausibility issues in the SACT, LOT, and mortality data could cause faulty determination of treatment discontinuation date and status of censoring.

The fact that Data set B included a lower percentage of patients receiving the target SACT (237/4003, 5.9% vs 1200/4808, 24.96% in Data set A) limited the utility of the data for determining the rwTTD. This finding highlights the need and importance of conducting a rigorous and use case–specific data quality assessment in the planning stage of RWD studies. In addition, for Data set B, findings of extremely low and high gaps between target SACT administration dates would warrant further investigation of each patient’s trajectory to verify the specific data quality issue before taking proper data quality improvement actions such as removing the patient or the SACT record as outliers.

### Limitations

This study has several limitations that require further discussion. First, adequately assessing the reasons for missingness across different RWD sources is challenging. In particular, the data feeds and capture of elements across different data sources are variable. A lack of transparency and consistency means that different RWD sources are often not fully interoperable [38]. In this study, we applied cohort attrition steps to align populations represented in the 2 data sets and imputed the LOT end date and LOT name that were missing in Data set B. However, a major remaining roadblock was the vendor’s privacy-preserving aggregation, which does not allow data sources to be adequately reviewed on more granular level to understand the reason behind missing data, data quality issues, or data discrepancies.

Second, the implementation of data quality checks for new RWD sources, especially for those with data table structures that differ from those of prior data sets, requires customization and reconfiguration that are often time consuming. We are developing a data dashboard tool that can accelerate this process for both raw data and a common data model such as that of the Observational Health Data Sciences and Informatics [17,18].

Third, use case–specific data quality assessment checks often provide only a limited view of the comparative validity of the RWD under consideration, particularly when a well-recognized gold standard is absent. The paucity of data often limits an effective comparison with the distribution of key data elements in the general population (external validity). In this study, we set a priori metrics for these checks by using domain knowledge such as HNC prevalence [33] and regulatory approval timelines. It would be interesting for future studies to validate and update these metrics.

### Comparison With Prior Work

Prior studies have evaluated rwTTD, also known as the duration of therapy and real-world time on treatment, for immuno-oncology agents used in treating recurrent or metastatic HNC [39], advanced non-small cell lung cancer [28,40-42], and other solid cancers [42]. In contrast to this study, these studies drew on research-ready databases (as would be identified in the preassessment step of UReQA [3]), and the actions taken to ensure RWD fitness and quality were limited to aligning patient eligibility criteria (the cohort definition step of UReQA [3]).

New use cases can be created for other medication-related outcomes or therapeutic areas by following the first 3 steps of implementing the rwTTD use case in this study. In addition, the data quality checks that we identified and created for the rwTTD use case can be used for other types of use cases. For example, checks on medication identification and dates can also be used to evaluate the fitness of RWD sources for studying medication adherence. The checks on mortality and follow-up visits could validate the applicability of an RWD source for survival analyses.

### Future Work

We selected 2 US EHR-based oncology databases to implement the UReQA use case of rwTTD. These were the only 2 databases the research team had access to that provided both oncology treatment and LOT information during the time of study execution. Each database may have its own bias in representing the overall advanced HNC population in the United States. Future work could implement (1) evaluation of more US EHR-based oncology databases to bring more impactful findings and (2) investigating the associations between rwTTD calculation and quantitative data quality assessment for various medications of interest and cancer types.

### Conclusions

The fit-for-purpose quality assessment demonstrated the high level of variability in quality of the 2 real-world data sets for estimating the rwTTD of an SACT for advanced HNC. This study illustrates the application and value of use case–specific data assessment tasks in identifying high-quality RWD for research studies. The data quality specifications supporting this comprehensive use case can be expanded to other use cases in oncology outcomes research. Incorporating such comprehensive data quality assessment could help the study team select the most suitable database in the planning stage of a real-world evidence study. In addition, understanding data quality concerns particularly relevant to research questions can provide additional insights for properly preparing data in full study execution.

## Appendix

Multimedia Appendix 1 Data checks comprising 20 tasks assessing conformance, completeness, or plausibility.

## Abbreviations

EHR: electronic health record
HNC: head and neck cancer
ICD: International Classification of Diseases
LOT: line of therapy
Q3: third quarter
RWD: real-world data
rwTTD: real-world time to treatment discontinuation
SACT: systemic anticancer therapy
UReQA: Use Case Specific Relevance and Quality Assessment

## Data Availability

The data sets generated during and/or analyzed during this study are not publicly available as they were data vendors’ proprietary assets provided to the study team under commercial licenses but are available from the corresponding author on reasonable request and permission from the data vendor.

In addition, we cannot disclose the identities of the data vendors, as doing so would inevitably promote the business of 1 data vendor and may violate data use agreements.
