# Supplementary material for: Real-World Data Quality Framework for Oncology Time to Treatment Discontinuation Use Case: Implementation and Evaluation Study
Source: JMIR Med Inform. 2024 Mar 6;12:e47744. doi: 10.2196/47744 (PMC10955397; doi:10.2196/47744)
Supplement: Multimedia Appendix 1 [file medinform_v12i1e47744_app1.docx]

**Multimedia Appendix 1.** Data checks comprising 20 tasks assessing conformance, completeness, or plausibility [13].^a^

| Task # | Data check, including subtask | Category |
| --- | --- | --- |
|  |  |  |
| **1** |  |  |
|  | N (%) of patients with a recorded administration or order for any SACT^b^ after the prespecified study index date^c^ | Completeness |
| **2** |  |  |
|  | % of SACT drug records with missing drug identity information (name and code) | Completeness |
|  | - Check if SACT drug name has been normalized - Check length of NDC^d^   Check if RxNorm code is at ingredient level or drug level | Conformance |
| **3** |  |  |
|  | N (%) of patients with target SACT^e^ administration date after the study index date | Completeness |
| **4** |  |  |
|  | N (%) of SACT drug administration records with complete administration date | Completeness |
| **5** |  |  |
|  | Distribution of gaps (in days) between target SACT drug administration dates | Plausibility |
| **6** |  |  |
|  | N (%) of patients with target SACT order date after the study index date | Completeness |
| **7** |  |  |
|  | N (%) of all SACT drug order records with complete refill information | Completeness |
| **8** |  |  |
|  | N (%) of all SACT drug order records with complete order date | Completeness |
| **9** |  |  |
|  | Distribution of gaps (in days) between target SACT drug order dates, normalized by refill and cancellation record | Plausibility |
| **10** |  |  |
|  | Number of patients who initiated the target SACT by year and quarter^f^ | Plausibility |
| **11** |  |  |
|  | Completeness of information regarding LOT^g^ number, LOT name, LOT start date, LOT end date | Completeness |
| **12** |  |  |
|  | N (%) of patients for whom the first LOT number after the study index date is not 1 | Plausibility |
| **13** |  |  |
|  | Distribution of LOT number for initiation of target SACT | Plausibility |
| **14** |  |  |
|  | Use of target SACT in a specified LOT before the approval date | Plausibility |
| **15** |  |  |
|  | N (%) of patients with a death record | Completeness |
| **16** |  |  |
|  | N (%) of patients with multiple records of different death dates | Plausibility |
| **17** |  |  |
|  | N (%) of patients with records showing healthcare activity after the death date | Plausibility |
| **18** |  |  |
|  | Unique number of patients and count of unique patient-date pairs in inpatient, outpatient, pharmacy records for each type of clinical data after the study index date | Completeness |
| **19** |  |  |
|  | Distribution of number of visits between the first and last date of target SACT administration or order, normalized by the time window between first and last target SACT date | Completeness |
| **20** |  |  |
|  | Distribution of gap (in days) between date of the last visit and date of last administration or use of target SACT | Completeness |

^a^The background colors correspond to each of the four operational steps (steps 1 to 4) to ascertain rwTTD, as depicted in Table 1.

^b^SACT: systemic anticancer therapy.

^c^For this study, the index date was the date of first advanced head and neck cancer diagnosis.

^d^NDC: National Drug Code.

^e^For this study, the target SACT was an immunotherapy drug administered intravenously for advanced head and neck cancer.

^f^Quarter (Q) refers to the division of each years into fourths: Q1, January to March; Q2, April to June; Q3, July to September; Q4, October to December.

^g^LOT: line of therapy.
